# Clinical mastitis in ewes; bacteriology, epidemiology and clinical features

**DOI:** 10.1186/1751-0147-49-23

**Published:** 2007-09-24

**Authors:** Tormod Mørk, Steinar Waage, Tore Tollersrud, Bjørg Kvitle, Ståle Sviland

**Affiliations:** 1 Department of Animal Health, National Veterinary Institute, PO Box 8156 Dep, N-0033 Oslo, Norway; 2 Department of Production Animal Clinical Sciences, Norwegian School of Veterinary Science, PO Box 8146 Dep, N-0033 Oslo, Norway

## Abstract

**Background:**

Clinical mastitis is an important disease in sheep. The objective of this work was to identify causal bacteria and study certain epidemiological and clinical features of clinical mastitis in ewes kept for meat and wool production.

**Methods:**

The study included 509 ewes with clinical mastitis from 353 flocks located in 14 of the 19 counties in Norway. Clinical examination and collection of udder secretions were carried out by veterinarians. Pulsed-field gel electrophoresis (PFGE) was performed on 92 *Staphylococcus aureus *isolates from 64 ewes.

**Results and conclusion:**

*S. aureus *was recovered from 65.3% of 547 clinically affected mammary glands, coagulase-negative staphylococci from 2.9%, enterobacteria, mainly *Escherichia coli*, from 7.3%, *Streptococcus *spp. from 4.6%, *Mannheimia haemolytica *from 1.8% and various other bacteria from 4.9%, while no bacteria were cultured from 13.2% of the samples. Forty percent of the ewes with unilateral clinical *S. aureus *mastitis also had a subclinical *S. aureus *infection in the other mammary gland. Twenty-four of 28 (86%) pairs of *S. aureus *isolates obtained from clinically and subclinically affected mammary glands of the same ewe were indistinguishable by PFGE. The number of identical pairs was significantly greater than expected, based on the distribution of different *S. aureus *types within the flocks. One-third of the cases occurred during the first week after lambing, while a second peak was observed in the third week of lactation. Gangrene was present in 8.8% of the clinically affected glands; *S. aureus *was recovered from 72.9%, *Clostridium perfringens *from 6.3% and *E. coli *from 6.3% of the secretions from such glands. This study shows that *S. aureus *predominates as a cause of clinical ovine mastitis in Norway, also in very severe cases. Results also indicate that *S. aureus *is frequently spread between udder halves of infected ewes.

## Background

Mastitis is an important disease in sheep. Clinical cases are often severe; systemic signs are present and the condition is obviously painful. Clinically affected glands frequently suffer partial or complete damage and do not resume normal function. Reduced milk yield leads to decreased growth of the lambs [1-3]. Additional losses associated with clinical mastitis are costs of treatment and culling of ewes due to permanent udder damage [3-7]. In very severe cases, gangrene may develop in the mammary gland and the ewe may die. Thus, mastitis has a major impact on both economy and animal welfare in sheep production.

Although a wide range of microorganisms may cause ovine mastitis, most cases are reported to be due to staphylococci [8]. Several reports indicate that coagulase-negative staphylococci (CNS) are the most common cause of subclinical mastitis in dairy ewes [9-14], while both CNS and *Staphylococcus aureus *are frequent causes in meat sheep [5,15,16]. With regard to organisms associated with clinical mastitis, there are fewer reports published. *S. aureus *has been reported to be the most common causal organism in both meat [5,15,17-19] and dairy ewes [13,20,21]. *Mannheimia haemolytica *[5,18,19,22], *Escherichia coli *[13,18,19] and various streptococci [15,18,19] are other important causative organisms.

Differences in climate, production forms and management practices may give rise to differences both in the epidemiology, bacteriology and clinical manifestations of mastitis. In Norway, sheep are kept exclusively for meat and wool production. They are housed during the winter and early spring, including the lambing season.

The objective of this study was to identify bacteria associated with clinical ovine mastitis in Norway. In addition, certain epidemiological and clinical features of the disease were studied.

## Methods

### Animals and clinical data

**Figure 1:**
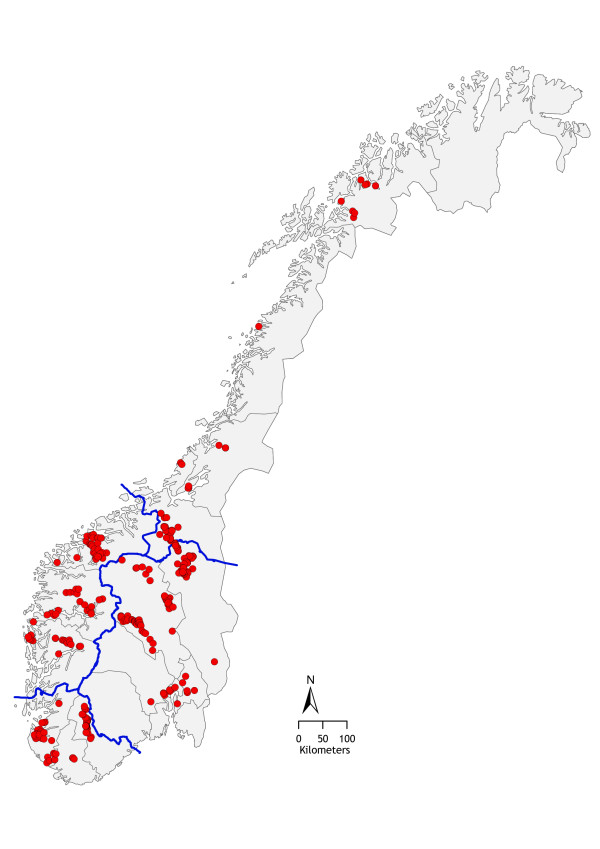
Map of Norway showing the location of the sheep flocks from which cases of clinical mastitis were obtained. Thin lines show county boundaries and thick lines region boundaries.

Udder secretions were collected and clinical data recorded from 509 ewes with clinical mastitis. The ewes belonged to 353 flocks located in 14 counties in Norway (Figure 1). The geographical distribution of the cases is shown in Table 1. Clinical mastitis was present in one gland in 471 ewes and in both glands in 38 ewes. The study was carried out in 2002, 2003 and 2004. Only cases that occurred between 1 week prepartum and 8 weeks postpartum were included. In Norway, lambing generally takes place in April and May.

**Table 1:** Distribution by region and county of 547 milk samples obtained from ovine mammary glands with clinical mastitis, and of the 509^a ^ewes and 353 flocks from which the samples originated.

Region	County	No. of flocks	No. of ewes	No. of glands
East	Akershus	13	27	31
	Hedmark	68	121	128
	Oppland	60	85	86
	Buskerud	4	5	6
South	Aust-Agder	34	56	57
	Vest-Agder	4	6	7
	Rogaland	40	48	53
West	Hordaland	22	26	28
	Sogn og Fjordane	25	25	29
	Møre og Romsdal	32	39	40
North	Sør-Trøndelag	33	46	50
	Nord-Trøndelag	6	10	13
	Nordland	3	3	4
	Troms	9	12	15

^a ^Four hundred and seventy-one ewes with unilateral and 38 with bilateral intramammary infection.

### Data and sample collection

Thirty-two veterinary practitioners contributed to the study. When called to a case of clinical mastitis, the veterinarian was to examine the ewe, collect udder secretions and record information regarding the identity, age, date of parturition, the number of lambs and the clinical condition of the ewe on a standardized form. Clinical data included the rectal temperature, an assessment of the severity of systemic signs (graded as none, weak, moderate or severe) and local clinical signs of the affected gland, including whether or not gangrene was present (i.e., cold and blue udder and teat skin).

Prior to treatment, samples were collected aseptically from the clinically affected glands in 10-ml sterile plastic vials by the veterinary practitioner according to the International Dairy Federation's standards [23]. Additionally, samples were taken from the clinically unaffected gland of 252 of the ewes with unilateral clinical mastitis. The samples were sent by mail to the laboratory as soon as possible after sampling, or frozen and stored at -20°C until submission.

If a ewe experienced more than one episode of mastitis during the observation period, only the first episode was included in the study.

### Microbiological methods

The samples were examined at the National Veterinary Institute or at the TINE Mastitis Laboratory in Molde, Norway, and bacteria were identified according to the recommendations of the International Dairy Federation [23] with additions. The National Veterinary Institute and the TINE Mastitis Laboratory are both quality assured in accordance with NS-EN ISO/IEC 17025. Briefly, the secretions were brought to room temperature, assessed visually and characterized by appearance before they were mechanically shaken and 10 μl plated on Bacto Blood Agar Base No 2 (Difco Laboratories, Detroit, MI, USA) containing 5% washed bovine erythrocytes and incubated for 48 hours in a 5% CO_2 _atmosphere at 37°C. Cultures were read at 24 and 48 hours. If growth was not detected after incubation for 24 hours, the original sample was incubated for 4 hours at 37°C and 50 μl aliquots plated and incubated for 24 hours under aerobic (5% CO_2 _atmosphere) and anaerobic conditions.

Bacterial species were identified tentatively by their gross colony morphology and by Gram staining, and further confirmatory tests were used as necessary. All suspected staphylococcal colonies were tested using the tube coagulase test (Becton Dickinson Microbiology Systems, Bedford, MA, USA). Coagulase-positive staphylococci were streaked on peptone agar (p-agar) (Difco, Sparks, MD) supplemented with 7 mg/l of acriflavin (Sigma-Aldrich Chemie, Steinheim, Germany) [24], and incubated at 37°C for 24 hours. Bacterial growth in the full length of the streak on p-agar was considered confirmative of *S. aureus*. Isolates identified as *S. aureus *were stored at -70°C in Bacto Heart Infusion Broth (Difco) with 15% glycerol. *E. coli *was identified by the lactose and indole tests, and other enterobacteria were identified to the species or genus level by using a microtube identification system (API 20 E^®^; bioMérieux S.A., Marcy-l'Etoile, France). *Streptococcus uberis*, *Streptococcus dysgalactiae*, *Streptococcus agalactiae*, *Streptococcus *spp. and *Enterococcus *spp. were distinguished by the CAMP reaction, the aesculin and inulin tests and by culture on the bromthymolblue lactose-sucrose agar. Bacteria within the family *Pasteurellaceae *were identified to the species level by the CAMP reaction, the indole, mannitol, sorbitol, trehalose, dulcitol, oxidase and beta-galactosidase tests and the haemolysis patterns. *Clostridium perfringens *was differentiated from other *Clostridium *spp. by colony morphology, immobility and the presence of a zone of partial haemolysis and a zone of complete haemolysis. *Arcanobacterium pyogenes *was identified by Gram staining and the presence of pinpoint colonies surrounded by a narrow zone of clear haemolysis at 48 hours. None of the samples were from ewes with arthritis, conjunctivitis or pneumonia; therefore, the mammary secretions were not checked for the presence of mycoplasms.

Total DNA was prepared and pulsed-field gel electrophoresis (PFGE) performed as described previously [25] on 92 *S. aureus *isolates from 21 flocks in which at least one ewe had bilateral *S. aureus *intramammary infection (IMI) and where at least two ewes experienced clinical *S. aureus *mastitis. The band patterns were compared visually. Isolates with indistinguishable patterns were considered identical PFGE types while those with at least one band difference were considered to be different types.

### Statistical methods

The chi-square test was used to compare the frequencies of cases within different time intervals in relation to parturition and the relative proportions of clinical *S. aureus *cases and gangrenous mastitis cases in ewes of different parity and with different number of lambs.

The distribution of pairs of *S. aureus *PFGE types within flocks (equal vs. unequal) in ewes with bilateral IMI was compared with the corresponding distribution that would be expected if all *S. aureus *isolates found within each flock were paired randomly. All isolates from the flocks that supplied two or more cases of clinical *S. aureus *mastitis, of which at least one ewe had bilateral IMI, were included, and Fisher's exact test was used to test the probability of identical distributions of the observed and expected pairs.

*P *< 0.05 was considered statistically significant.

## Results

### Bacteriological and epidemiological findings

The distribution of bacteria cultured from secretions from the glands with clinical mastitis is shown in Table 2. *S. aureus *was the predominant pathogen and was found in 65.3% of the samples from affected glands. In the samples from the southern, eastern, western and northern regions, *S. aureus *was found in 76.0%, 59.0%, 63.9% and 69.5%, respectively.

**Table 2:** Results of culture of secretions recovered from 547 mammary glands with clinical mastitis.

Bacteriological finding	n	%
*Staphylococcus aureus*	357	65.3
Coagulase-negative staphylococci	16	2.9
*Streptococcus uberis*	9	1.6
*Streptococcus dysgalactiae*^*a*^	8	1.5
*Streptococcus *spp.^*b*^	8	1.5
*Enterococcus *spp.	4	0.7
*Escherichia coli*	35	6.4
*Klebsiella pneumoniae*	2	0.4
*Enterobacter *spp.	3	0.5
*Mannheimia haemolytica*	10	1.8
*Arcanobacterium pyogenes*	4	0.7
*Clostridium perfringens*	7	1.3
*Pasteurella *spp.^*c*^	4	0.7
No growth	72	13.2
Contaminated samples^*d*^	8	1.5

^*a *^Subsp. *dysgalactiae*.

^*b *^Other than *Str. dysgalactiae *and *Str. uberis*.

^*c*^* Pasteurella mairii *(2 samples), *Pasteurella multocida *(2 samples).

^*d *^If blood agar plates contained more than two different types of colonies.

**Figure 2:**
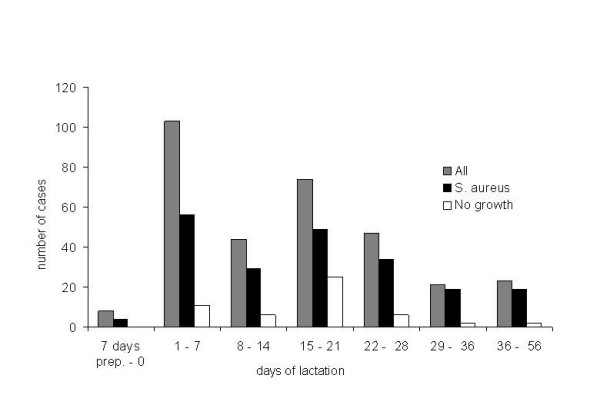
Distribution of 318 cases of clinical mastitis in relation to weeks of lactation.

Information about the date of parturition was received from 318 of the 471 cases of unilateral clinical mastitis. The distribution of the observed clinical mastitis cases in relation to the time of parturition is shown in Figure 2. The relative proportion of cases was greatest during the first week after lambing. Sixty-four (20.1%) of the 318 ewes for which the times of lambing and treatment were recorded, were treated for clinical mastitis during the first two days after parturition. The proportion of cases was significantly greater in the first (*P *< 0.005) and the third (*P *< 0.05) week postpartum as compared with the second week postpartum.

The proportion of the clinical *S. aureus *cases among all the clinical cases did not differ significantly between weeks of lactation, between ewes of different parities or between ewes with different number of lambs (data not shown).

In 180 ewes with unilateral clinical *S. aureus *mastitis, from which samples from both mammary glands were examined, *S. aureus *was found in secretions from 72 (40.0%) of the glands without clinical signs. In 72 ewes with unilateral clinical mastitis not caused by *S. aureus*, a subclinical *S. aureus *infection was found to be present in 10 (13.9%) of the glands without clinical signs.

### *S. aureus *PFGE types in ewes with bilateral IMI

A total of 22 different PFGE types were found among 92 *S. aureus *isolates from 21 farms. Comparisons of *S. aureus *pairs revealed that 24 (86.0%) of 28 pairs had indistinguishable band patterns (Table 3). Given the PFGE types of all *S. aureus *isolates within each flock, expected flock-specific distributions of PFGE type pairs based on all possible random pairwise combinations of isolates were arranged. The number of pairs with identical types observed in the flocks was significantly greater than that expected, when assuming a random distribution of isolates (*P *< 0.0001).

**Table 3:** Distribution within flocks of 22 different PFGE types of 92 *S. aureus *isolates from ewes with intramammary infection (IMI). Only flocks in which at least one ewe experienced bilateral *S. aureus *IMI and two ewes experienced clinical mastitis are included. The observed pairs of PFGE types from the ewes with bilateral *S. aureus *IMI are shown. Also shown is the expected distribution of pairs with equal and unequal PFGE type combinations when assuming random pairwise distribution of the observed isolates within each flock.

				Expected pairs (n)	
				
Flock	Ewes with IMI (n)	*S. auerus *types (n) present in flock	Bilateral PFGE type combinations	Equal	Unequal
F1	6	K, P, Q (4), R	QQ	6	15
F2	9	I, J, L (7), M	LL	21	24
F3	2	R (3)	RR	3	0
F4	3	R (4)	RR	6	0
F5	2	H (2), O (2)	HH, OO	2	4
F6	2	D (2), H	DD	1	2
F7	2	H, I, U	IU	0	3
F8	2	H (3), X	HH, HX	3	3
F9	2	D, H (3)	DH, HH	3	3
F10	2	I, M, V	IV	0	3
F11	6	H (5), I, W	HH	10	11
F12	3	H, I (3)	II	3	3
F13	3	H (4), I (2)	HH, HH, II	7	8
F14	3	F, J (3)	JJ	3	3
F15	2	H, S (2)	SS	1	2
F16	2	D (3)	DD	3	0
F17	4	H (2), I (2), N, G (2)	HH, II, GG	3	18
F18	2	C (2), E	CC	1	2
F19	2	C (2), G	CC	1	2
F20	3	I (2), R, T	II	1	5
F21	2	A (2), B	AA	1	2

### Clinical signs

Of the 471 cases of unilateral clinical mastitis systemic signs were recorded in 325 (Table 4) and the rectal temperature was measured in 342. Moderate or severe systemic signs were present in 159 (48.9%) ewes and 193 (56.4%) ewes had a rectal temperature above 40.0°C. Thirty-seven (11.8%) out of 313 ewes showed no systemic signs and had a rectal temperature below 40.1°C.

**Table 4:** Distribution of 325 of the 471 ewes with unilateral clinical mastitis by causal organism and the systemic signs^*a*^.

Pathogen	1 No. (%)	2 No. (%)	3 No. (%)	4 No. (%)	Total
All	51 (15.7)	115 (35.4)	119 (36.6)	40 (12.3)	325
*S. aureus*	30 (13.5)	77 (34.5)	89 (39.9)	27 (12.1)	223
Enterobacteria	2 (7.7)	9 (34.6)	11 (42.3)	4 (15.4)	26
No growth	11 (34.4)	12 (37.5)	4 (12.5)	5 (15.6)	32

^*a *^1 = no systemic signs, 2 = weak systemic signs, 3 = moderate systemic signs, 4 = severe systemic signs.

Gangrene was present in 48 (8.8%) of 547 clinically affected udder halves and *S. aureus *was found in 35 (72.9%), *C. perfringens *in 3 (6.3%), *E. coli *in 3 (6.3%) and *A. pyogenes *in 1 (2.1%). Bacteria were not found in 4 (8.3%) samples from such cases and two samples were contaminated. The degree of systemic influence was recorded in 33 ewes with gangrenous mastitis. Thirty (90.9%) of these cases exhibited moderate or severe signs. The proportion of the gangrenous mastitis cases among all the clinical cases did not differ significantly between weeks of lactation, between ewes of different parities or between ewes with different number of lambs (data not shown).

## Discussion

A random and representative selection of sheep flocks was not deemed feasible for this type of study. However, in order to have a reasonably representative geographical spread of the mastitis cases ewes from 14 of the 19 counties in Norway were included. Thus, the present flocks were housed and pastured under various conditions, and the variations in climatic conditions, flock size and management routines of Norwegian sheep production were reasonably well represented. During the lambing season and subsequent weeks the ewes are paid close attention. Later, most flocks are moved to pastures in the forests or mountains. For this reason, the study was restricted to cases that occurred between 1 week prepartum and 8 weeks postpartum in order to obtain clinical cases of recent origin.

*S. aureus *was found in 65% of the samples from clinically affected glands. The dominance of *S. aureus *as a cause of clinical ovine mastitis has also been shown in regional studies in Norway, where *S. aureus *was isolated from udder secretions of between 64 and 87% of the ewes [26,27]. In the present study, the largest proportion of *S. aureus *was found in the southern region (76%). A similar proportion of such cases (75%) was previously observed in a study including cases from one of the municipalities of this region [28]. Studies of clinical mastitis in meat sheep in other countries have found varying, though mostly relatively great, proportions of *S. aureus *infections (20–60%) [5,17-20,22]. In a study of dairy sheep in Jordan, Lafi et al. [13] found that 22% of the clinical mastitis cases were caused by *S. aureus*.

The main *S. aureus *reservoirs in sheep are suggested to be infected mammary glands and teat lesions [29]. However, *S. aureus *can also be cultured from intact teat skin and other body sites [10,30,31]. In dairy flocks, transfer during milking is considered an important mechanism for the spread of this organism [29]. In flocks of meat sheep, transmission of *S. aureus *between ewes could be a result of the herdsman transmitting *S. aureus *between ewes during manual udder control, or the udder being exposed to bedding material contaminated from infected ewes [6,7]. Some lambs occasionally suck other ewes than their dam, which might be a mechanism for the spread of *S. aureus *[32]. In Norway, routine examination of teats and udders are performed after weaning, and ewes with palpable abnormalities or which have experienced clinical mastitis are usually slaughtered before the breeding season. This contributes to decreasing the reservoir of *S. aureus*, but it obviously does not eliminate it.

Studies in other countries have reported prevalences of subclinical *S. aureus *IMI to be between 1 and 6% [12,13,15,33]. This indicates that subclinically infected glands are an important reservoir of *S. aureus *that can only be detected through bacteriological examination. In this study, 14% of the ewes with unilateral clinical mastitis caused by other pathogens than *S. aureus *had a subclinical *S. aureus *infection in the other mammary gland, while 40% of the ewes with clinical *S. aureus *infection in one gland had a subclinical *S. aureus *infection in the other. PFGE typing showed that 86% of the pairs of isolates from ewes with bilateral *S. aureus *IMI were indistinguishable. This percentage was much higher than what would be expected if the isolates from each of the flocks were paired at random, thus demonstrating a considerably greater tendency for spread of *S. aureus *between the udder halves of a ewe than between ewes within a flock.

The very low percentage of *M. haemolytica *in the clinically affected glands in this study (1.8%) contrasts results of clinical mastitis surveys in meat sheep in the UK and Ireland, where the proportions of cases caused by this organism were found to be approximately 50% and 21%, respectively [5,18]. Enterobacteria, mainly *E. coli*, were obtained from 7.3% of the clinically affected udder halves, which is similar to the proportions found in other studies on meat sheep [5,18,22]. However, the number of clinical cases caused by Gram negative bacteria and *A. pyogenes *may be underestimated because the samples were frozen before bacteriological analysis [34,35].

It is noteworthy that nearly 85% of the ewes exhibited systemic signs and that gangrene was present in as much as 9% of the clinically affected udder halves, clearly showing that ovine clinical mastitis must be considered a serious animal welfare problem. However, mild cases of clinical mastitis are most likely underrepresented in this study. According to Norwegian legislation, antibiotic treatment of animals must be initiated by a veterinarian and, for economical reasons, farmers might be reluctant to call for a veterinary surgeon to treat mild clinical cases.

Most mastitis cases occurred close to lambing. One-third of the ewes developed clinical mastitis during the first week after lambing, and a second peak, although somewhat smaller, was observed in the third week postpartum. This is in accordance with data from the Norwegian Sheep Recording System [36]. Likewise, studies in the UK and Ireland found that cases of acute clinical mastitis occurred most frequently during the first week of lactation, while a second peak occurred between the third and fourth week [5] or the fourth and seventh week [18] after lambing. Clinical cases around parturition might be newly acquired IMI or aggravations of existing subclinical infections [37]. The proportion of very severe cases, in which gangrene had developed, was not greater among cases occurring close to lambing as compared with those occurring later. The second peak could be explained by increased milk demand from the lambs and the eruption of incisors, which increases the risk of teat lesions. It has been reported that teat lesions frequently are present in ewes with clinical mastitis three to four weeks after lambing [38].

## Conclusion

This study shows that *S. aureus *is the most common cause of clinical mastitis in sheep in Norway and that this organism is frequently spread between glands in infected ewes. Further studies identifying predisposing factors, including reservoirs, transmission routes and factors facilitating *S. aureus *infection of the ovine mammary gland, are needed in order to improve strategies to reduce the occurrence of mastitis in sheep.

## Competing interests

The author(s) declare that they have no competing interests.

## Authors' contributions

TM, SW, TT and SS have been involved in the design of the study and the protocols. TM has been responsible for the field project. BK, TM and SS have performed most of the microbiological work in the laboratory. BK has performed the PFGE. SW and SS have been responsible for data analysis in cooperation with TM and TT. TM drafted the manuscript, but all the authors have contributed substantially to the final manuscript. All authors have read and approved the final manuscript.
